# Strain-specific metastatic phenotypes in pheochromocytoma allograft mice

**DOI:** 10.1530/ERC-18-0136

**Published:** 2018-07-12

**Authors:** Martin Ullrich, Josephine Liers, Mirko Peitzsch, Anja Feldmann, Ralf Bergmann, Ulrich Sommer, Susan Richter, Stefan R Bornstein, Michael Bachmann, Graeme Eisenhofer, Christian G Ziegler, Jens Pietzsch

**Affiliations:** 1 Department of Radiopharmaceutical and Chemical BiologyHelmholtz-Zentrum Dresden-Rossendorf, Institute of Radiopharmaceutical Cancer Research, Dresden, Germany; 2 Technische Universität DresdenSchool of Medicine, Faculty of Medicine Carl Gustav Carus, Dresden, Germany; 3 Technische Universität DresdenUniversity Hospital Carl Gustav Carus, Institute of Clinical Chemistry and Laboratory Medicine, Dresden, Germany; 4 Department of RadioimmunologyHelmholtz-Zentrum Dresden-Rossendorf, Institute of Radiopharmaceutical Cancer Research, Dresden, Germany; 5 Technische Universität DresdenUniversity Hospital Carl Gustav Carus, Institute of Pathology, Dresden, Germany; 6 Department of Internal Medicine IIITechnische Universität Dresden, University Hospital Carl Gustav Carus, Dresden, Germany; 7 Technische Universität DresdenUniversity Hospital Carl Gustav Carus, Universitäts Krebs Centrum (UCC), Tumorimmunology, Dresden, Germany; 8 Technische Universität DresdenNational Center for Tumor Diseases (NCT), Dresden, Germany; 9 Technische Universität DresdenSchool of Science, Faculty of Chemistry and Food Chemistry, Dresden, Germany

**Keywords:** neuroendocrine tumors, catecholamines, bioluminescence imaging, somatostatin receptors, small animal positron emission tomography, metastasis

## Abstract

Somatostatin receptor-targeting endoradiotherapy offers potential for treating metastatic pheochromocytomas and paragangliomas, an approach likely to benefit from combination radiosensitization therapy. To provide reliable preclinical *in vivo* models of metastatic disease, this study characterized the metastatic spread of luciferase-expressing mouse pheochromocytoma (MPC) cells in mouse strains with different immunologic conditions. Bioluminescence imaging showed that, in contrast to subcutaneous non-metastatic engraftment of luciferase-expressing MPC cells in NMRI-nude mice, intravenous cell injection provided only suboptimal metastatic spread in both NMRI-nude mice and hairless SCID (SHO) mice. Treatment of NMRI-nude mice with anti-Asialo GM1 serum enhanced metastatic spread due to substantial depletion of natural killer (NK) cells. However, reproducible metastatic spread was only observed in NK cell-defective SCID/beige mice and in hairless immunocompetent SKH1 mice bearing disseminated or liver metastases, respectively. Liquid chromatography tandem mass spectrometry of urine samples showed that subcutaneous and metastasized tumor models exhibit comparable renal monoamine excretion profiles characterized by increasing urinary dopamine, 3-methoxytyramine, norepinephrine and normetanephrine. Metastases-related epinephrine and metanephrine were only detectable in SCID/beige mice. Positron emission tomography and immunohistochemistry revealed that all metastases maintained somatostatin receptor-specific radiotracer uptake and immunoreactivity, respectively. In conclusion, we demonstrate that intravenous injection of luciferase-expressing MPC cells into SCID/beige and SKH1 mice provides reproducible and clinically relevant spread of catecholamine-producing and somatostatin receptor-positive metastases. These standardized preclinical models allow for precise monitoring of disease progression and should facilitate further investigations on theranostic approaches against metastatic pheochromocytomas and paragangliomas.

## Introduction

Adrenal pheochromocytomas and extra adrenal paragangliomas (PPGLs) are rare catecholamine-producing tumors of chromaffin cell origin (Lenders* et al.* 2005, Harari & Inabnet 2011, Jemal* et al.* 2011). In contrast to most other neoplasms, at least 30% of PPGLs have a hereditary background with variable development of metastatic disease dependent on the mutated gene (Lenders* et al.* 2005). Germline mutations in succinate dehydrogenase subunit B (*SDHB*) are associated with a particularly high penetrance of metastatic disease (Amar* et al.* 2007). Metastasizing PPGL cells disseminate via lymphatics and blood stream and give rise to solid organ metastases mainly in lymph nodes, bones, lungs and liver (Salmenkivi* et al.* 2004).

Commonly recommended treatment options for metastatic PPGLs include surgery for removing the tumor bulk, different combinations of chemotherapy, endoradiotherapy using [^131^I]metaiodobenzylguanidine, external radiation therapy to areas such as bone where metastases are not accessible for surgery, embolization to block tumor blood supply and sometimes also cryo- or radiofrequency ablation. However, these treatment options are often considered as palliative (PDQ® Adult Treatment Editorial Board).

Furthermore, somatostatin type 2 receptor (SSTR2)-targeting endoradiotherapy offers potential for treating metastatic PPGLs, using e.g. [^177^Lu]Lu-DOTA-(Tyr^3^)octreotate ([^177^Lu]Lu-DOTA-TATE) (Ullrich* et al.* 2016, Kong *et al.* 2017). Since response rates are usually between 30 and 60% (Castinetti* et al.* 2015), this approach is likely to benefit from combination with adjuvant, for example, radiosensitization therapy. Due to the complexity of the metastatic disease, investigations on additional, e.g., adjuvant and radiosensitizing treatments should be performed using preclinical *in vivo* models reproducibly providing tumors at clinically related metastatic sites.

Currently, no fully differentiated human PPGL cell line model is available for mirroring human disease in mice. An available chromaffin progenitor cell line has been established from a primary human pheochromocytoma but does not produce catecholamines, limiting its utility as a chromaffin cell model (Ghayee* et al.* 2013). Alternatively, the mouse pheochromocytoma (MPC) cell line deriving from an adrenal tumor of a neurofibromin 1-knockout mouse (Powers* et al.* 2000) provides an appropriate model for preclinical investigations on metastatic PPGLs *in vitro* and* in vivo* (Korpershoek* et al.* 2012, Ziegler* et al.* 2013).

From native MPC cells, a subcutaneous tumor model has initially been generated *in vivo* using NCr-nude mice (NU(NCr)-*Foxn1^nu^*) reproducibly providing non-metastatic allografts (Ohta* et al.* 2006, 2008). Furthermore, also NMRI-nude mice (Rj:NMRI-*Foxn1^nu^*) have been successfully employed for generating a standardized subcutaneous tumor model from genetically engineered MPC^mCherry^ cells (Ullrich* et al.* 2014). This particular model resembles at least in part biochemical features and molecular characteristics of sporadic and hereditary human PPGLs and allows for monitoring tumor progression and treatment response using both *in vivo* fluorescence imaging and measurement of catecholamines in urine (Ullrich* et al.* 2016).

The first generation of metastatic pheochromocytoma allograft models was based on intravenous tumor cell injection into immunodeficient NCr-nude mice (NU(NCr)-*Foxn1^nu^*) (Ohta* et al.* 2006, Martiniova* et al.* 2009, Giubellino* et al.* 2012). These models provided metastasized allografts occurring predominantly in the liver but only rarely in other organs. It is known that the immunologic phenotype and the general genetic background of mice substantially influence the metastatic spread of circulating tumor cells (Khanna & Hunter 2005). We therefore hypothesized that intravenous injection of MPC cells into mouse strains featuring different immunologic phenotypes provides allograft models showing a more reproducible pattern of clinically related metastases.

To address the above hypothesis, our objective was to characterize the metastatic spread of luciferase-expressing MPC cells after intravenous injection in mouse strains featuring different immunologic phenotypes and to compare tumor progression, catecholamine excretion and SSTR2 status of different metastases models with a previously established subcutaneous reference model (NMRI-nude mice) (Ullrich* et al.* 2014, 2016). For non-invasive detection of organ metastases in murine pheochromocytoma models, MRI and bioluminescence imaging (BLI) provide appropriate sensitivity *in vivo* (Martiniova* et al.* 2011, Giubellino* et al.* 2012). To quantify MPC tumor burden, measurement of urinary monoamines provides comparable sensitivity to preclinical imaging as has been demonstrated by us in a subcutaneous allograft model (Ullrich* et al.* 2014). In the same model, positron emission tomography (PET) using radiolabeled somatostatin analogs allowed for functional imaging of SSTR2 (Ullrich* et al.* 2016).

Using the aforementioned tools, two mouse strains (SCID/beige and SKH1) were identified providing a highly reproducible and clinically relevant pattern of metastasized allografts that can be precisely monitored. These models should facilitate preclinical investigations on theranostic approaches against metastatic PPGLs and help to further understand biological features of these tumors.

## Materials and methods

### Cell culture and luciferase gene transfer

Mouse pheochromocytoma cells (MPCs clone 4/30PRR (Powers* et al.* 2000)) were routinely cultured as described previously (Ullrich* et al.* 2014). MPC cells (passage 32) were genetically modified using the lentiviral transfer vector p6NST50-luc (Supplementary Fig. 1, see section on supplementary data given at the end of this article) harboring the open reading frame of a firefly luciferase (*luc*) and a combined expression cassette of enhanced green-fluorescent protein and zeocin resistance (*egfp*-*zeo*) (Morgenroth* et al.* 2007, Stirnnagel* et al.* 2010, Ho* et al.* 2012). Genetically modified cells were named MPC^LUC/eGFP-ZEO^ (abbreviated as MPC^LUC/GZ^). Selection of genetically modified cells was performed using 100 µg/mL zeocin (Thermo Fisher Scientific). Cultures containing >90% of genetically modified cells were used for experiments (Supplementary Fig. 2A and B).

### Animal experiment

Animal experiments were carried out at the Helmholtz-Zentrum Dresden-Rossendorf according to the guidelines of German Regulations for Animal Welfare and have been approved by the Local Animal Ethics Committee for Animal Experiments (Landesdirektion Dresden, Germany). Female mice, between 10 and 14 weeks old, were housed in a pathogen-free facility. For tumor induction, an injectable suspension containing 2 × 10^6^ MPC^LUC/GZ^ cells in phosphate-buffered saline was prepared. For generation of the subcutaneous reference model a single bolus of 40 µL cell suspension was injected into the right shoulder of NMRI-nude mice (Rj:NMRI-*Foxn1^nu^*, T cell-deficient, hairless; Janvier Labs, Le Genest-Saint-Isle, France; *n* = 10). For generation of metastases models, a single bolus of 100 µL cell suspension was injected intravenously into the tail vein of NMRI-nude mice (*n* = 10), natural killer (NK) cell-depleted NMRI-nude mice (*n* = 5), SHO mice (Crl:SHO-*Prkdc^scid^ Hr^Hr^*, T and B cell-deficient, hairless; Charles River Laboratories; *n* = 9), SCID/beige mice (CB17.Cg-*Prkdc^scid^Lyst^bg-J^*/Crl, T and B cell-deficient, NK cell-defective, white-haired; Charles River; *n* = 9) and SKH1 mice (Crl:SKH1-Elite-*Hr^hr^*, immunocompetent, hairless; Charles River; *n* = 15). Anesthesia was induced and maintained with inhalation of 10% (v/v) desflurane (Baxter, Unterschleißheim, Germany) in 30% (v/v) oxygen air. Animals were killed using CO_2_ inhalation and cervical dislocation. For histologic examination, tumors and organs were excised and fixed using 4% (w/v) paraformaldehyde in Dulbecco’s phosphate-buffered saline containing 2% (w/v) sucrose. Bone samples were decalcified for 3 days in decalcifier soft (Carl Roth, Karlsruhe, Germany).

### NK cell-depleting pre-treatment of NMRI-nude mice

For depletion of NK cells in NMRI-nude mice, animals (*n* = 5) were pre-treated with 30 µL of anti-Asialo GM1 serum (Wako Chemicals GmbH, Neuss, Germany) diluted in Dulbecco’s phosphate-buffered saline and administered intraperitoneally 24 h before intravenous tumor cell injection. Treatment was repeated 2, 6, 9, 13 and 16 days after tumor cell injection.

### Bioluminescence imaging

BLI was performed using the *In vivo* Xtreme system (Bruker, Billerica, MA, USA). d-Luciferin was prepared and administered according to the manufacturer’s instructions (Caliper Life Sciences, Hopkinton, MA, USA). Images were analyzed using the MI 7.2 software (Bruker). *In vitro*, net luminescence intensity (photons/s/mm^2^) of serially diluted tumor cells was determined in a 96-well microplate (*n* = 2). *In vivo*, net luminescence intensities were determined from dynamic image series. Signals from successfully induced subcutaneous tumors in NMRI-nude mice (*n* = 10) were quantified from sagittal view images. Signals from successfully induced metastases in NMRI-nude (*n* = 7), NK cell-depleted NMRI-nude (*n* = 5), SHO (*n* = 6), SCID/beige (*n* = 9) and SKH1 mice (*n* = 12) were quantified by summating the intensities of ventral and dorsal view images. X-ray images were merged with bioluminescence images using the linear dodge blending mode of Photoshop CS5 (Adobe).

### Positron emission tomography

PET was performed as described previously (Ullrich* et al.* 2016) using the preclinical nanoSCAN PET/CT scanner (Mediso, Münster, Germany). Tumor-bearing SCID/beige mice (*n* = 2) and SKH1 mice (*n* = 2) received 15 MBq of [^68^Ga]Ga-DOTA-(Tyr^3^)octreotide (TOC) or [^68^Ga]Ga-DOTA-(Tyr^3^)octreotate (TATE) at a molar activity of 25 GBq/µmol, respectively. Images were merged with X-ray CT.

### Determination of free urinary monoamines

Single-voided urine samples of NMRI-nude mice bearing subcutaneous tumors (*n* = 10), of SCID/beige mice bearing disseminated metastases (*n* = 9) and of SKH1 mice bearing liver metastases (*n* = 7) were collected and urinary concentrations of monoamines were determined simultaneously using liquid chromatography tandem mass spectrometry as described elsewhere (Peitzsch* et al.* 2013, Ullrich* et al.* 2014).

### SSTR2 immunohistochemistry

SSTR2 immunohistochemistry was performed on paraformaldehyde-fixed paraffin-embedded tissue samples from SCID/beige mice bearing disseminated metastases (*n* = 3) and from SKH1 mice bearing liver metastases (*n* = 3) using the anti-SSTR2 (UMB1) primary antibody (Abcam) as described previously (Ullrich* et al.* 2016).

### Statistical analysis

Statistical analysis was performed using Prism, version 5.02 (GraphPad Software). Data are presented as mean ± standard error of the mean and *n* represents the number of data sets investigated. Exponential tumor progression curves were fitted from luminescence intensity data using the least squares method weighted by 1/*y*^2^. Time of tumor formation (when luminescence intensity of tumors exceeded the luminescence intensity of initially injected tumor cells) and tumor growth rates (% growth per day) were calculated from fitted data. Significance of differences was tested using ANOVA applying Holm–Šídák’s *post hoc* multiple comparison test. Differences were considered significant at *P* values <0.05. Significance of relationships was tested using Pearson’s linear correlation test and displayed as Pearson’s correlation coefficient (*r*_p_).

## Results

### BLI of MPC^LUC/GZ^ cells *in vitro* and initial cell distribution *in vivo*

**Figure 1:**
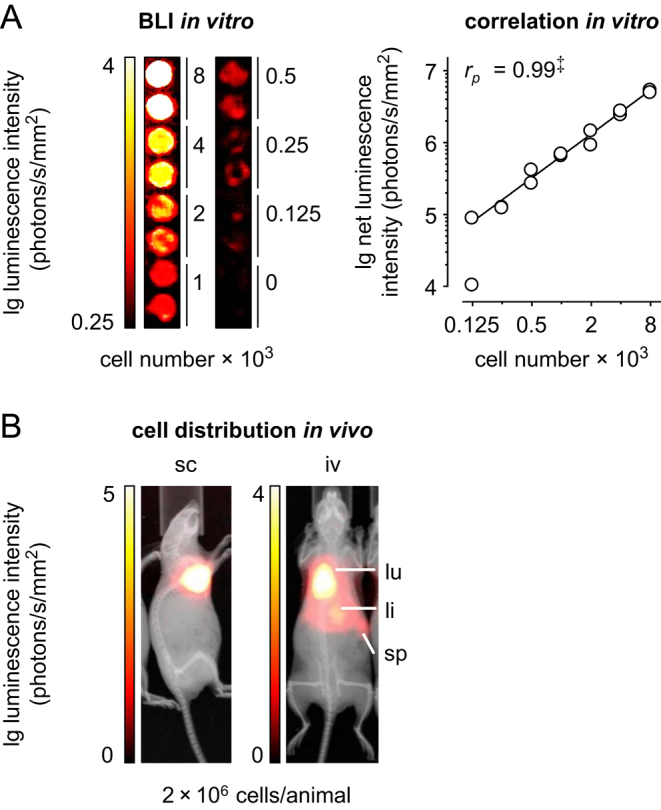
BLI of MPC^LUC/GZ^ cells *in vitro* and *in vivo*; (A) imaging sensitivity and correlation between number and luminescence intensity of serially diluted MPC^LUC/GZ^ cells in a 96-well microplate; *n* = 2; significance of relationship: ^‡^*P* < 0.001; (B) distribution of MPC^LUC/GZ^ cells in NMRI-nude mice 20 min after subcutaneous vs intravenous injection; (iv) intravenous, (li) liver, (lu) lung, (sc) subcutaneous, (sp) spleen. A full colour version of this figure is available at https://doi.org/10.1530/ERC-18-0136.

Luciferase-expressing mouse pheochromocytoma (MPC^LUC/GZ^) cells were generated (Supplementary Figs 1, 2A and B) to perform non-invasive detection of tumor formation in mice. *In vitro*, BLI detected a minimum of 125 tumor cells seeded in a 96-well microplate (Fig. 1A). Correlation analysis showed a significant positive linear relationship (*r*_p_ = 0.99) between number and luminescence intensity of tumor cells indicating that BLI allows for precise tumor cell quantification.

As exemplary shown for the NMRI-nude strain, *in vivo* BLI detected a local accumulation of subcutaneously injected MPC^LUC/GZ^ cells restricted to the injection site in the right shoulder (Fig. 1B, reference model). In the same strain, accumulation of intravenously injected MPC^LUC/GZ^ cells was detectable in lungs, liver and spleen (Fig. 1B). A comparable distribution of intravenously injected MPC^LUC/GZ^ cells was also observed in NK cell-depleted NMRI-nude, SHO, SCID/beige and SKH1 mice with slightly different dynamics (Supplementary Fig. 3A and B).

### Engraftment of MPC^LUC/GZ^ cells and metastatic spread in different mouse strains

Subcutaneous injection of MPC^LUC/GZ^ cells into our reference model, NMRI-nude mice, resulted in an engraftment rate of 100%. Intravenous tumor cell injection resulted in engraftment rates of 70% in NMRI-nude mice, 100% in NK cell-depleted NMRI-nude mice, 67% in SHO mice, 100% in SCID/beige mice and 80% in SKH1 mice (Table 1).

**Table 1:** Strain-specific characteristics of MPC^LUC/GZ^ allograft mice.

Investigations performed	NMRI-nude	NMRI-nude	NMRI-nude	SHO	SCID/beige	SKH1
Tumor cell injection route	Sc	iv	iv	iv	iv	iv
Anti-Asialo GM1 pre-treatment	No	No	Yes	No	No	No
*In vivo* BLI and analyses of luminescence intensities						
Initial tumor cell distribution	None	Lungs, liver, spleen	Lungs, liver, spleen (s.m.)	Lungs, liver, spleen (s.m.)	Lungs, liver, spleen (s.m.)	Lungs, liver, spleen (s.m.)
Metastatic phenotype	None	Scattered	Scattered	Scattered	Disseminated	Liver
Engraftment rate (*n*_en_/*n*_in_)	10/10	7/10	5/5	6/9	9/9	12/15
Time of tumor formation (day)	15 ± 1.4	38 ± 4.1^‡a^	32 ± 3.9^†a^	39 ± 7.8^‡1^	1.3 ± 0.7^†a^*^b^	19 ± 1.5
Tumor growth rate (%/day)	17 ± 1.4	22 ± 2.5	28 ± 2.5	19 ± 4.1	18 ± 0.8	40 ± 3.6^‡a^*^b^
Documentation of metastatic sites (*n*_me_/*n*_en_) identified from animal preparation and BLI						
Liver	0/10	1/7	3/5	3/6	8/9	12/12
Lungs	0/10	2/7	3/5	1/6	8/9	0/12
Adrenals	0/10	2/7	4/5	1/6	9/9	0/12
Ovaries	0/10	1/7	4/5	2/6	7/9	0/12
Bones	0/10	4/7	1/5	3/6	9/9	1/12
Lymph nodes	0/10	0/7	0/5	2/6	1/9	1/12
Peritoneum	0/10	1/7	2/5	2/6	7/9	4/12
Brain	0/10	1/7	4/5	1/6	2/9	0/12
Spleen	0/10	0/7	0/5	0/6	0/9	0/12
Pancreas	0/10	0/7	0/5	0/6	0/9	0/12
Detailed documentation of tumor pathology and histopathology						
Pathology	p.d.	n.a.	n.a.	n.a.	(s.m.)	(s.m.)
Histopathology	p.d.	n.a.	n.a.	n.a.	(s.m.)	(s.m.)
Tumor-related changes in urinary monoamine concentrations and correlation (*r*_p_) with luminescence intensities of tumors						
Dopamine	↑ (0.51)	n.a.	n.a.	n.a.	↑ (0.89^†^)	↑ (0.91^†^)
Norepinephrine	↑ (0.94^‡^)	n.a.	n.a.	n.a.	↑ (0.65)	↑ (0.87*)
Epinephrine	→ (0.16)	n.a.	n.a.	n.a.	↑ (0.74*)	→ (0.21)
3-Methoxytyramine	↑ (0.74*)	n.a.	n.a.	n.a.	↑ (0.80^†^)	↑ (0.94^†^)
Normetanephrine	↑ (0.87^‡^)	n.a.	n.a.	n.a.	↑ (0.71*)	↑ (0.91^†^)
Metanephrine	→ (0.64)	n.a.	n.a.	n.a.	↑ (0.83^†^)	↑ (0.78*)
Somatostatin type 2 receptor status of tumors as determined from *in vivo* PET imaging and immunohistochemistry						
Radiotracer uptake	p.d.	n.a.	n.a.	n.a.	^68^Ga-DOTA-TOC	^68^Ga-DOTA-TATE
Immunoreactivity	p.d.	n.a.	n.a.	n.a.	Positive	Positive
Validation of tumor tissue identification and BLI-based tumor quantification						
*Ex vivo* BLI of metastases	n.a.	n.a.	n.a.	n.a.	(s.m.)	n.a.
Correlation (*r*_p_) MRI vs BLI	(0.77^‡^) (s.m.)	n.a.	n.a.	n.a.	n.a.	(0.82^‡^) (s.m.)

(n.a.) not assessed; (p.d.) previously described in a subcutaneous MPC^mCherry^ allograft model by Ullrich *et al.* (2014, 2016); (s.m.) results included in supplemental materials; significance of differences or relationships: *P < 0.05, ^†^*P* < 0.01, ^‡^*P* < 0.001; ^a^differences compared to the subcutaneous NMRI-nude reference model; ^b^differences compared to every other intravenously induced metastases model; (iv) intravenous, (sc) subcutaneous; (*n*_en_) number of animals that showed successful tumor cell engraftment; (*n*_in_) number of animals that received tumor cell injection; (*n*_me_) number of animals that showed metastases at specified organs; tumor-related changes in renal monoamine excretion compared to basal levels before tumor cell injection: (↑) increased, (→) unchanged.

**Figure 2:**
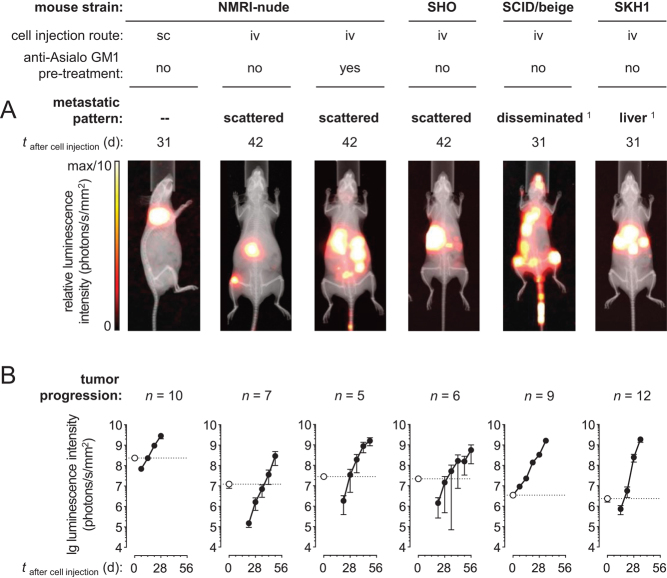
BLI of subcutaneous and metastasized MPC^LUC/GZ^ allografts in mice featuring different immunologic phenotypes; (A) metastatic spread at defined time points after cell injection; of note, images were individually scaled to 1/10 of the maximal luminescence intensity to also visualize small lesions, therefore, signal dimensions do not represent tumor size; (B) progression of luminescence intensities of subcutaneous tumors and metastases *in vivo*; logarithmic scaling of *y*-axis; (white data points, dotted lines) luminescence intensity of initially distributed tumor cells detected 32 min after subcutaneous and 20 min after intravenous cell injection (black data points, continuous lines) luminescence intensities of progressing tumors; depletion of natural killer (NK) cells resulted from treatment with anti-Asialo GM1 serum; the meaning of *scattered* metastases and *disseminated* metastases is given in the results section; (iv) intravenous, (sc) subcutaneous; ^1^animal models showing reproducible pattern of metastases. A full colour version of this figure is available at https://doi.org/10.1530/ERC-18-0136.

*In vivo*, BLI of MPC^LUC/GZ^ allografts (Fig. 2A) showed that subcutaneous cell injection into NMRI-nude mice induced ectopic non-metastasizing tumors. Intravenous cell injection was associated with a non-consistent pattern of sporadically *scattered* metastases in untreated NMRI-nude mice, NK cell-depleted NMRI-nude mice and SHO mice. In contrast, intravenous tumor cell injection into SCID/beige mice provided a consistent pattern of multifocal *disseminated* metastases in liver, adrenal glands, bones, lungs, ovaries, and, rarely, also in brain and attached to the peritoneum (Supplementary Figs 4A, B, C, D, E, F, G, H, 5A, B, C, D, E, F, G, H, I and 6A, B, C, D, E, F). Furthermore, intravenous tumor cell injection into SKH1 mice predominantly induced multifocal liver metastases (Supplementary Fig. 7A, B and C).

### Progression of metastasized MPC^LUC/GZ^ allografts in different mouse strains

Correlation analyses (Supplementary Fig. 8A, B, C and D) showed a significant positive linear relationship between tumor volume as determined using MRI (Supplementary information for methodologic details and discussion) and luminescence intensity of subcutaneous MPC^LUC/GZ^ tumors in NMRI-nude mice (*r*_p_ = 0.77) as well as between tumor volume and luminescence intensity of liver metastases in SKH1 mice (*r*_p_ = 0.82). These results indicate that BLI allows for semi-quantitative *in vivo* monitoring of MPC^LUC/GZ^ cell-derived subcutaneous tumors and metastases in mice with comparable accuracy.

Monitoring the luminescence intensities of progressing MPC^LUC/GZ^ allografts *in vivo* (Fig. 2B) showed that tumor formation (time when luminescence intensity of tumors exceeded the luminescence intensity of initially injected tumor cells) occurred 15 days after subcutaneous cell injection in NMRI-nude mice. After intravenous cell injection, formation of metastases was significantly delayed, requiring 38 days in untreated NMRI-nude mice, 32 days in NK cell-depleted NMRI-nude mice and 39 days in SHO mice. In contrast, formation of disseminated metastases already occurred 1.3 days after intravenous cell injection in SCID/beige mice even though these particular bioluminescence signals were most likely emitted from surviving tumor cells along the tail vein injection route. Liver metastases in SKH1 mice were detected 19 days after intravenous cell injection and showed a significantly higher exponential growth rate (40%/day) compared to the other metastases models (between 17 and 27%/day).

Initial BLI measurements showed that after intravenous MPC^LUC/GZ^ cell injection, NMRI-nude, NK cell-depleted NMRI-nude and SHO mice did not meet the requirements for reproducible induction of metastases. Therefore, further characterization of metastatic phenotypes was only carried out in SCID/beige and SKH1 mice and pathophysiology was compared to the subcutaneous NMRI-nude reference model.

### Renal monoamine excretion related to subcutaneous vs metastasized MPC^LUC/GZ^ allografts

**Figure 3:**
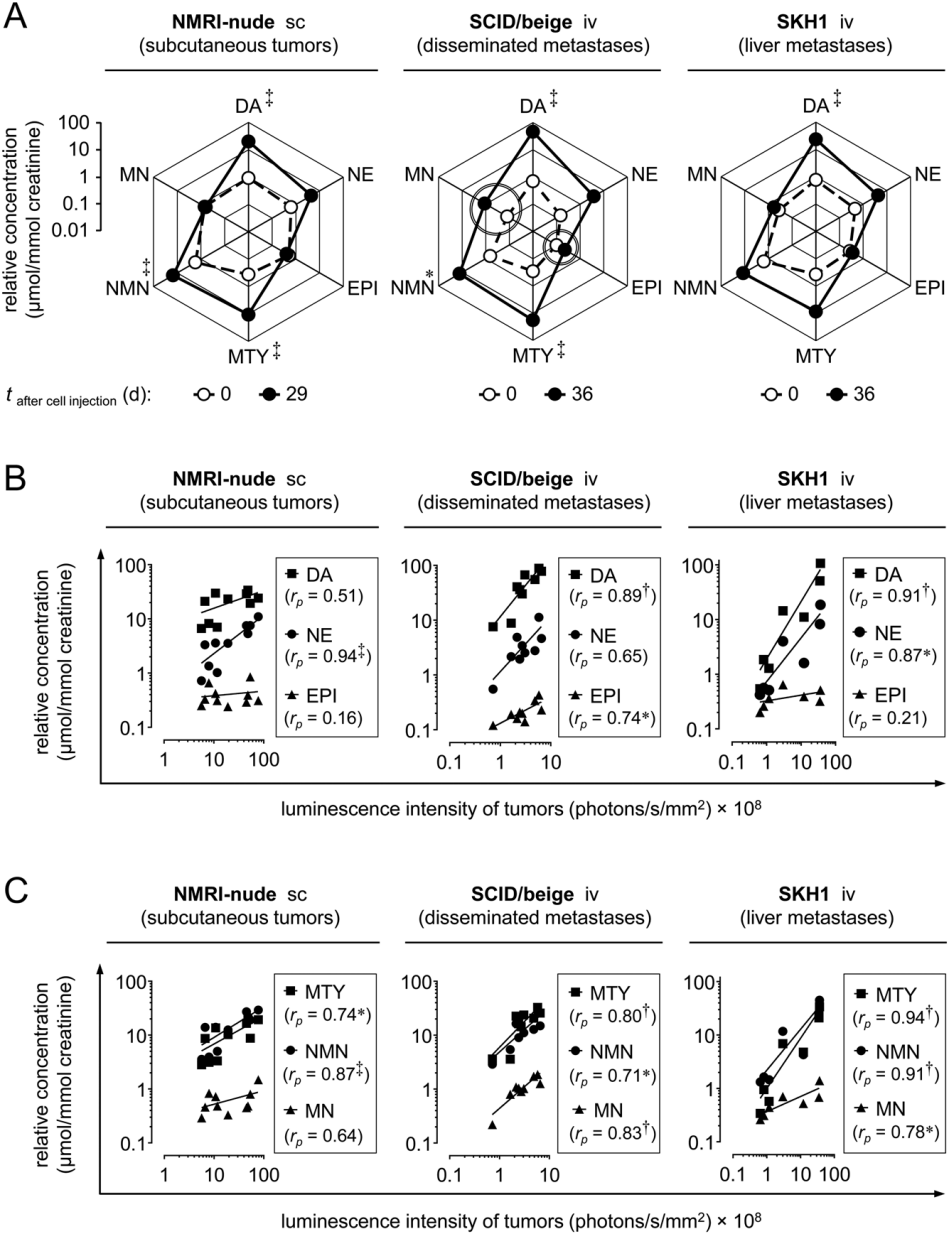
Renal monoamine excretion of subcutaneous and metastasized MPC^LUC/GZ^ allograft models; (A) pathophysiologic changes in monoamine excretion profiles related to subcutaneous tumors in NMRI-nude mice, disseminated metastases in SCID/beige mice and liver metastases in SKH1 mice; (white data points, dashed line) physiologic excretion profile before tumor cell injection; (black data points, continuous line) excretion profile after a defined time of tumor growth; double-lined circles mark increasing urinary concentrations of epinephrine and metanephrine detectable in SCID/beige mice; (B and C) correlation between luminescence intensity of stumors and urinary concentrations of free catecholamines and *O*-methylated catecholamine metabolites; logarithmic scaling of *x*- and *y*-axes; significance of differences or relationships: *P < 0.05, ^‡^*P* < 0.001; (DA) dopamine, (EPI) epinephrine, (MN) metanephrine, (MTY) 3-methoxytyramine, (NE) norepinephrine, (NMN) normetanephrine, (iv) intravenous, (sc) subcutaneous.

Measurement of urinary catecholamines and their respective *O*-methylated metabolites (Fig. 3A) revealed that subcutaneous MPC^LUC/GZ^ tumors in NMRI-nude mice, disseminated metastases in SCID/beige mice and liver metastases in SKH1 mice, were all associated with comparable changes in renal monoamine excretion profiles characterized by increasing concentrations of dopamine, norepinephrine, 3-methoxytyramine and normetanephrine. In SCID/beige mice, a significantly lower basal monoamine excretion exceptionally allowed for additional detection of small changes in metastases-related urinary epinephrine and metanephrine.

Correlation analyses (Fig. 3A, B and C) showed significant linear relationships between luminescence intensities of MPC^LUC/GZ^ tumors and urinary concentrations of three urinary monoamines in the subcutaneous NMRI-nude reference model (norepinephrine, 3-methoxytyramine, normetanephrine), compared to five urinary monoamines in the disseminated SCID/beige metastases model (dopamine, epinephrine, 3-methoxytyramine, normetanephrine, metanephrine) and five urinary monoamines in the SKH1 liver metastases model (dopamine, norepinephrine, 3-methoxytyramine, metanephrine, epinephrine). Of note, significant linear relationships with luminescence intensities of tumors were more frequently observed among *O*-methylated catecholamine metabolites compared to free catecholamines. These results indicate that measurement of urinary monoamines allows for non-invasive and precise quantification of metastasized MPC^LUC/GZ^ allografts in mice.

### Somatostatin type 2 receptors in metastasized MPC^LUC/GZ^ allografts

**Figure 4:**
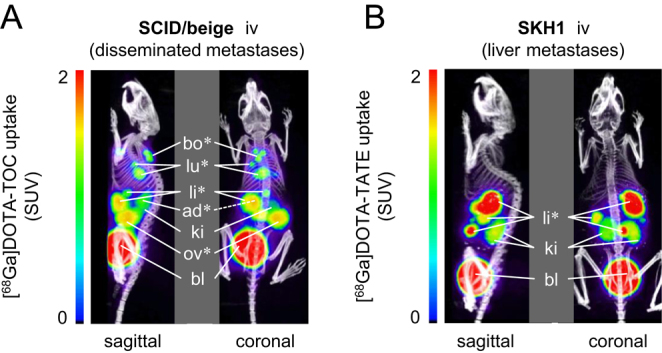
PET imaging of SSTR2 in two metastasized MPC^LUC/GZ^ allograft models; positron emission tomograms merged with X-ray computed tomograms as anatomic references; maximum intensity projections 90 min after radiotracer injection; (A) [^68^Ga]Ga-DOTA-TOC uptake of disseminated metastases in SCID/beige mice; (B) [^68^Ga]Ga-DOTA-TATE uptake of liver metastases in SKH1 mice; (*) metastatic sites; (ad) adrenal gland; (bl) bladder; (bo) bone, scapula; (ki) kidneys; (li) liver; (lu) lung; (ov) ovary; (SUV) standardized uptake value. A full colour version of this figure is available at https://doi.org/10.1530/ERC-18-0136.

PET showed specific uptake of the somatostatin type 2 receptor (SSTR2)-targeting radiotracer [^68^Ga]Ga-DOTA-TOC in MPC^LUC/GZ^ metastases in bone, lung, liver and ovaries of SCID/beige mice (Fig. 4A). A second radiotracer [^68^Ga]Ga-DOTA-TATE, which exhibits comparable SSTR2 specificity to [^68^Ga]Ga-DOTA-TOC, was specifically taken up by liver metastases in SKH1 mice (Fig. 4B).

**Figure 5:**
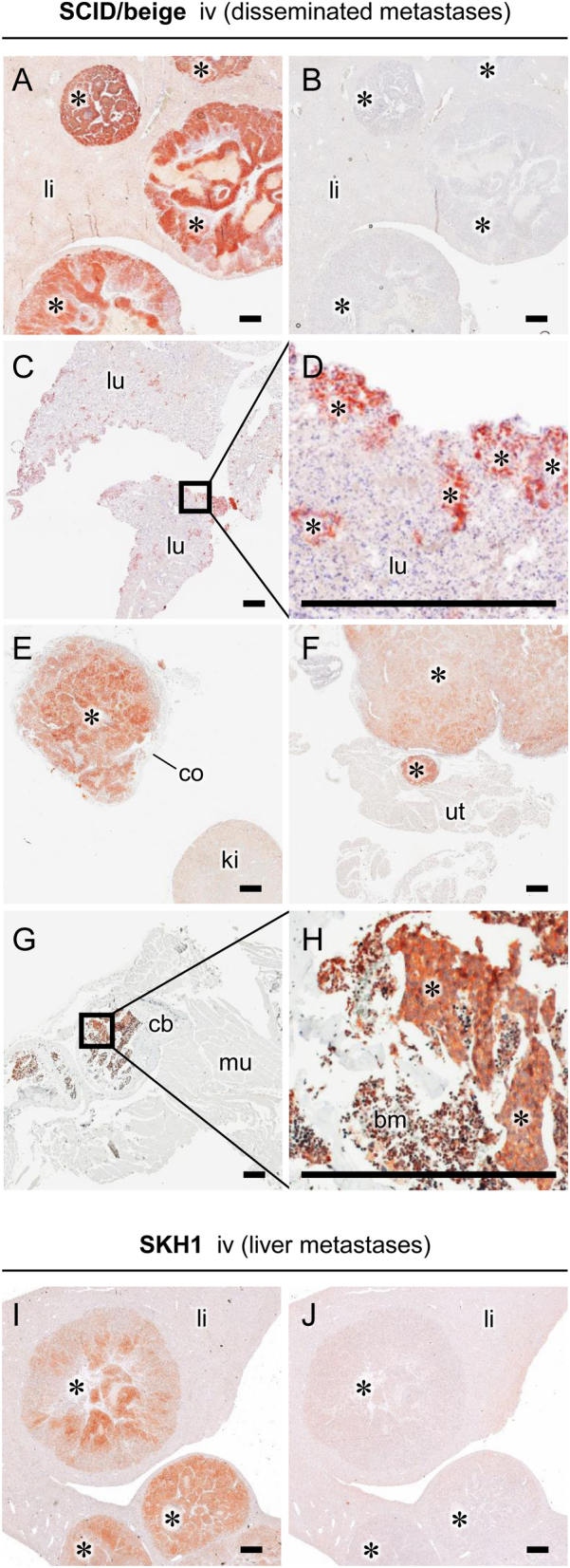
SSTR2 immunohistochemistry of metastasized MPC^LUC/GZ^ allografts in SCID/beige and SKH1 mice: (A, I) liver metastases showing initial necrosis; (B, J) IgG isotype control stain of liver metastases; (C, D) small scattered metastases from periphery of lung tissue; (E) adrenal metastasis surrounded by adrenocortical tissue; (F) ovarian metastasis connected to uterine tissue; (G, H) nests of bone metastases surrounded by bone marrow; (*) metastasized allografts; (bm) bone marrow; (cb) cortical bone; (co) adrenocortical tissue; (iv) intravenous, (ki) kidney; (li) liver tissue; (lu) lung tissue; (mu) muscle tissue; (sc) subcutaneous; (ut) uterine tissue; scale bar: 0.5 mm. A full colour version of this figure is available at https://doi.org/10.1530/ERC-18-0136.

Immunohistochemistry on metastasized MPC^LUC/GZ^ allografts from SCID/beige and SKH1 mice confirmed elevated SSTR2 levels in metastases compared to surrounding healthy tissue (Fig. 5A, B, C, D, E, F, G, H, I and J). These results indicate that SSTR2 is maintained in metastasized MPC^LUC/GZ^ allografts, irrespective of metastatic site or mouse strain, emphasizing its utility as a molecular theranostic target.

## Discussion

The present study characterized mouse strain-specific metastatic phenotypes of intravenously induced luciferase-expressing MPC^LUC/GZ^ allografts, in particular with regard to metastatic spread, tumor progression, catecholamine excretion and somatostatin type 2 (SSTR2) receptor status. The investigated mouse strains SKH1, NMRI-nude, SHO and SCID/beige were pre-selected with regard to increasing severity of genetically related immune cell defects according to manufacturer’s specifications and due to a hairless or white-haired phenotype for better optical imaging of organ metastases (for details see ‘Materials and methods’ section). In particular, intravenous injection of MPC^LUC/GZ^ cells into SCID/beige and SKH1 mice resulted in highly reproducible and clinically relevant pattern of catecholamine-producing and SSTR2-positive metastases. These models allow for monitoring the progression of metastases precisely by two methods, *in vivo* BLI and measurement of tumor-related urinary monoamines.

In a previous study, we investigated the efficiency of radiolabeled DOTA-(Tyr^3^)octreotate for SSTR2-targeted imaging ([^64^Cu]Cu-DOTA-TATE) and treatment ([^177^Lu]Lu-DOTA-TATE) of metastatic PPGLs in a subcutaneous MPC-mCherry allograft model using NMRI-nude mice (Ullrich* et al.* 2016). For this particular radiopharmacologic investigation, the subcutaneous tumor model had the advantage that the location of one single lesion was known and its size and morphology could easily be measured allowing for precise determination of radiotracer uptake and treatment response.

Nevertheless, subcutaneous MPC allografts do not entirely resemble the microenvironment at clinically relevant target organs of PPGL metastasis such as lymph nodes, bones, lungs and liver (Salmenkivi* et al.* 2004). In terms of modeling metastases *in vivo*, injection of tumor cells into the blood circulation of mice is a common approach. The difficulty thereafter is to monitor disease progression. For this purpose, the genetically modified luciferase-expressing MPC^LUC/GZ^ cell line was generated allowing for *in vivo* BLI of metastasized allografts after intravenous injection into a tail vein.

Using the intravenous cell injection route, it has to be considered that formation of metastasized allografts arising from circulating MPC^LUC/GZ^ cells does not depend on vascular invasion, pre-metastatic selection and epithelial–mesenchymal transition usually occurring in metastasizing primary tumors (Labelle & Hynes 2012, Daphu* et al.* 2013). Thus, these early metastatic events cannot be investigated in intravenously induced MPC^LUC/GZ^ metastases models. Formation of metastases in these models primarily depends on later metastatic events such as dissemination of MPC^LUC/GZ^ cells via lymph system or blood circulation, extravasation and interactions of tumor cells with blood components and target tissues.

This study demonstrates that metastatic phenotypes of intravenously induced MPC^LUC/GZ^ allograft models strongly depend on genetic background and immunologic condition of the mouse stain. After intravenous injection, MPC^LUC/GZ^ cells did not circulate long, with the majority accumulating rapidly in lungs and smaller fractions in liver and spleen. In contrast to this initial cell distribution, in particular lungs later showed a much lower metastatic burden compared to liver; spleen remained free from metastases. Our results indicate that the engraftment of circulating MPC^LUC/GZ^ cells in murine organs essentially depends on the tissue-specific microenvironment rather than on the number of entrapped cells.

NMRI-nude mice carrying a homozygous *Foxn1^nu^* mutation are frequently used for generating tumor allograft or xenograft models. These mice lack a thymus and are therefore T cell deficient allowing for the engraftment of tumor cells or tumor tissue without activation of adaptive immune responses. Since NMRI-nude mice have been successfully employed for generating a standardized subcutaneous tumor model from genetically engineered MPC^mCherry^ cells (Ullrich* et al.* 2014, 2016), we consequently investigated whether this particular strain would also allow for generating a standardized metastases model after intravenous injection of MPC^LUC/GZ^ cells. Under the investigated conditions, only subcutaneous cell injection allowed for reproducible engraftment of MPC^LUC/GZ^ cells in NMRI-nude mice, whereas intravenous cell injection led to inefficient engraftment and non-reproducible metastatic scatter. Since combined T and B cell deficiency in SHO mice carrying a homozygous *Prkdc^scid^* mutation did not improve the engraftment of circulating MPC^LUC/GZ^ cells, we suspect innate immune responses to be responsible.

NK cells are essential effectors in the response to tumor formation. Consistent with previous reports showing that the genetic background of NMRI-nude and SHO mice is associated with high NK cell numbers (Dorshkind* et al.* 1985, Budzynski & Radzikowski 1994), both strains not only showed suboptimal engraftment of circulating MPC^LUC/GZ^ cells, but also the longest delay in tumor formation. In support of our hypothesis, *in vivo* depletion of NK cells selectively performed in NMRI-nude mice using anti-Asialo GM1 serum facilitated the engraftment of circulating MPC^LUC/GZ^ cells and preponed tumor formation; however, number and activity of NK cells were not determined in these mice. Additional interactions of anti-Asialo GM1 serum with murine monocytes, fetal thymocytes and basophils might have also contributed (Habu* et al.* 1981, Kasai* et al.* 1981, Nishikado* et al.* 2011).

In SCID/beige mice, impaired NK cell function due to the *Lyst^bg-J^* mutation in addition to T and B cell deficiency (MacDougall* et al.* 1990) was associated with highly efficient engraftment of circulating MPC^LUC/GZ^ cells and almost immediate tumor formation. These findings indicate that NK cells are substantially involved in the innate immune response against circulating MPC^LUC/GZ^ cells. Thus, it appears that well-directed NK cell response against circulating tumor cells may allow for protective treatment against metastasizing PPGL cells.

The highly reproducible pattern of disseminated MPC^LUC/GZ^ metastases in SCID/beige mice provides an opportunity for studying metastasis to the bone, one of the most frequent target organs of metastatic PPGLs in patients. Furthermore, MPC^LUC/GZ^ metastases occurring in the adrenal glands of SCID/beige mice morphologically resemble orthotopic tumors, potentially enabling studies on primary pheochromocytomas, too.

SKH1 mice are capable of generating both innate and adaptive immune responses against circulating MPC^LUC/GZ^ cells. Interestingly, only liver tissue of SKH1 mice provided an adequate hosting microenvironment for successful engraftment of MPC^LUC/GZ^ cells presumably due to organ-specific immunologic tolerance or further unknown processes. Most importantly, MPC^LUC/GZ^ liver metastases in SKH1 mice prospectively allow for therapeutic investigations in the presence of an entirely functional immune system.

For BLI of metastasized pheochromocytoma allografts in mice, other genetically modified luciferase-expressing cell lines such as MPC-GL9 (Powers* et al.* 2014) and mouse tumor tissue (MTT)-luc (Giubellino* et al.* 2012) have been generated previously. However, MPC-GL9 cells lack an antibiotic resistance for selection *in vitro* and MTT-luc cell-derived tumors have shown uncommonly high aggressiveness *in vivo.* In contrast, zeocin resistance of the MPC^LUC/GZ^ cell line allowed for efficient selection of genetically modified clones *in vitro*. Moreover, MPC^LUC/GZ^ cell-derived metastases in mice progressed with reasonable aggressiveness and therefore provide an appropriate time frame for therapeutic testing.

The present study demonstrates that measurement of renal monoamine excretion in SCID/beige and SKH1 mice, in particular of dopamine, 3-methoxytyramine and normetanephrine, allows for most precise non-invasive quantification of metastasized MPC^LUC/GZ^ allografts. Thus, this preclinical diagnostic approach follows clinical procedures based on measurements of metanephrines in plasma and urine for detecting PPGLs (Lenders & Eisenhofer 2017).

Although MPC cells are known to express phenylethanolamine *N*-methyltransferase (PNMT), the key enzyme of epinephrine synthesis (Powers* et al.* 2000), considerable increases particularly in urinary epinephrine and its *O*-methylated metabolite metanephrine were only detectable in SCID/beige mice bearing disseminated MPC^LUC/GZ^ metastases. The prevalent appearance of adrenal metastases in SCID/beige mice may explain these observations since adrenal cortical steroids are known to induce PNMT in primary pheochromocytomas (Eisenhofer* et al.* 2011). Furthermore, a low basal monoamine excretion and a high metastatic burden in SCID/beige mice may also have facilitated the detection of tumor-related epinephrine and metanephrine in urine. Taken together, measurement of tumor-related monoamines in urine provides a reliable alternative to small animal imaging approaches in terms of monitoring the overall burden of metastasized MPC^LUC/GZ^ allografts in mice.

PET and immunohistochemistry showed that SSTR2 is a molecular theranostic target also in metastasized MPC^LUC/GZ^ allografts, irrespective of metastatic site or mouse strain. These findings are in agreement with clinical findings of high SSTR2 densities in metastatic human PPGLs, especially in those due to mutations in succinate dehydrogenase subunit 2 (*SDHB*) (Elston* et al.* 2015, Janssen* et al.* 2015, Tan* et al.* 2015). Thus, metastasized MPC^LUC/GZ^ allografts may be a predictor of metastatic lesion responses to SSTR2-targeting treatments.

In conclusion, this study demonstrates that intravenous injection of MPC^LUC/GZ^ cells into SCID/beige and SKH1 mice provides reproducible and clinically relevant pattern of catecholamine-producing and SSTR2-positive metastases. These preclinical models allow for monitoring disease progression precisely using BLI and measurements of tumor-related urinary monoamines. Prospectively, MPC^LUC/GZ^ metastases models will facilitate investigations on therapeutic approaches against metastatic PPGLs based on combinations of SSTR2-targeted endoradiotherapy with adjuvant and radiosensitizing drugs, for example, cyclooxygenase 2-selective inhibitors (Laube* et al.* 2016).

## Supplementary Material

Supporting Figure 1

Supporting Figure 2

Supporting Figure 3

Supporting Figure 4

Supporting Figure 5

Supporting Figure 6

Supporting Figure 7

Supporting Figure 8

Supporting Figure 9

Supplemental material

## Declaration of interest

The authors have declared that no competing interest exists. The funding sponsors had no role in the design of the study; in the collection, analyses, or interpretation of data; in the writing of the manuscript or in the decision to publish the results.

## Funding

This work was supported by the Deutsche Forschungsgemeinschaft (DFG) grants BE-2607/1-2 (R B and J P), ZI-1362/2-2 (C G Z and G E), the Collaborative Research Center Transregio 205 ‘*The Adrenal: Central Relay in Health and Disease*’ (CRC/TRR 205/1; M U, M P, S R, S R B, G E, C G Z, and J P) and the Paradifference Foundation (Consortium for Personalized Targeted Therapy for SDHB-mutated Metastatic PPGLs).

## Author contribution statement

M U and J P jointly conceived and supervised the study. M U, J L and R B performed experiments and analyzed data. M P performed liquid chromatography tandem mass spectrometry analyses. A F and M B installed the luciferase reporter gene in MPC cells. U S contributed histopathologic examinations. S R and C G Z contributed cell analyses. M B, G E and J P provided analytical tools and supported the supply of reagents. S R B, G E and M B gave conceptual and editorial advice. M U, C G Z and J P interpreted data and wrote the paper. All authors discussed results and implications and commented on the manuscript at all stages. All authors read the paper and contributed to its final form.
