# Supplementary figures and images for: Strain-specific metastatic phenotypes in pheochromocytoma allograft mice
Source: Endocr Relat Cancer. 2018 Jul 12;25(12):993–1004. doi: 10.1530/ERC-18-0136 (PMC6176113; doi:10.1530/ERC-18-0136)

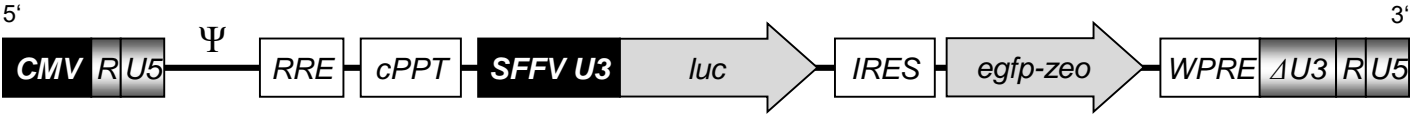

Supplemental Figure 1

Supplement: Supporting Figure 1 [file erc-25-993-s001.pdf]

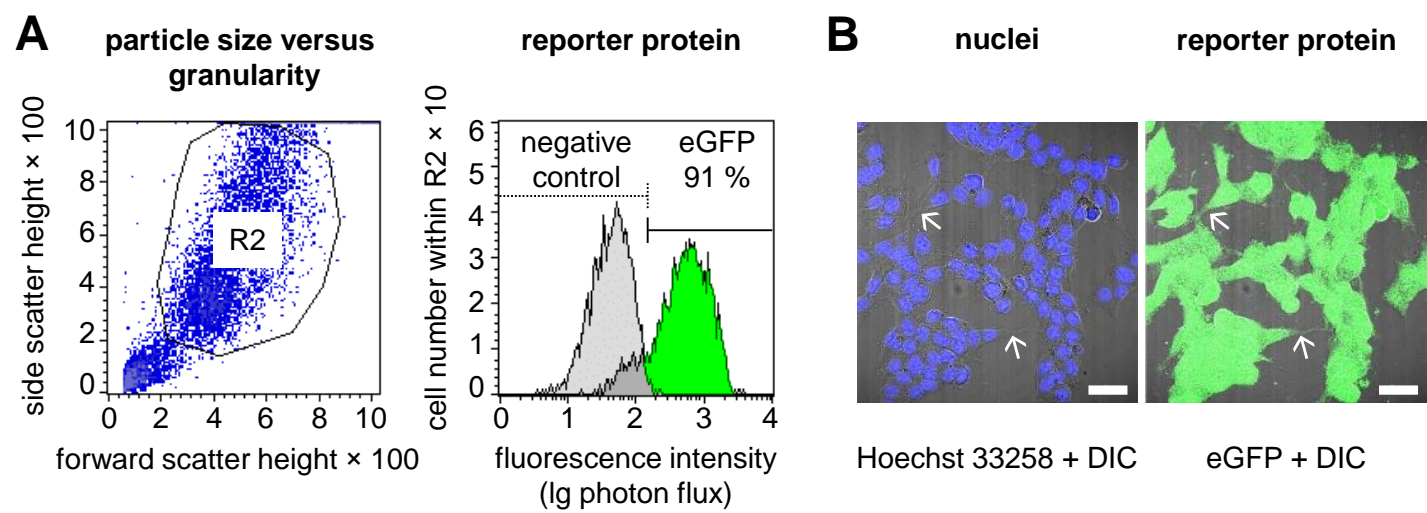

**Supplemental Figure 2**

Supplement: Supporting Figure 2 [file erc-25-993-s002.pdf]

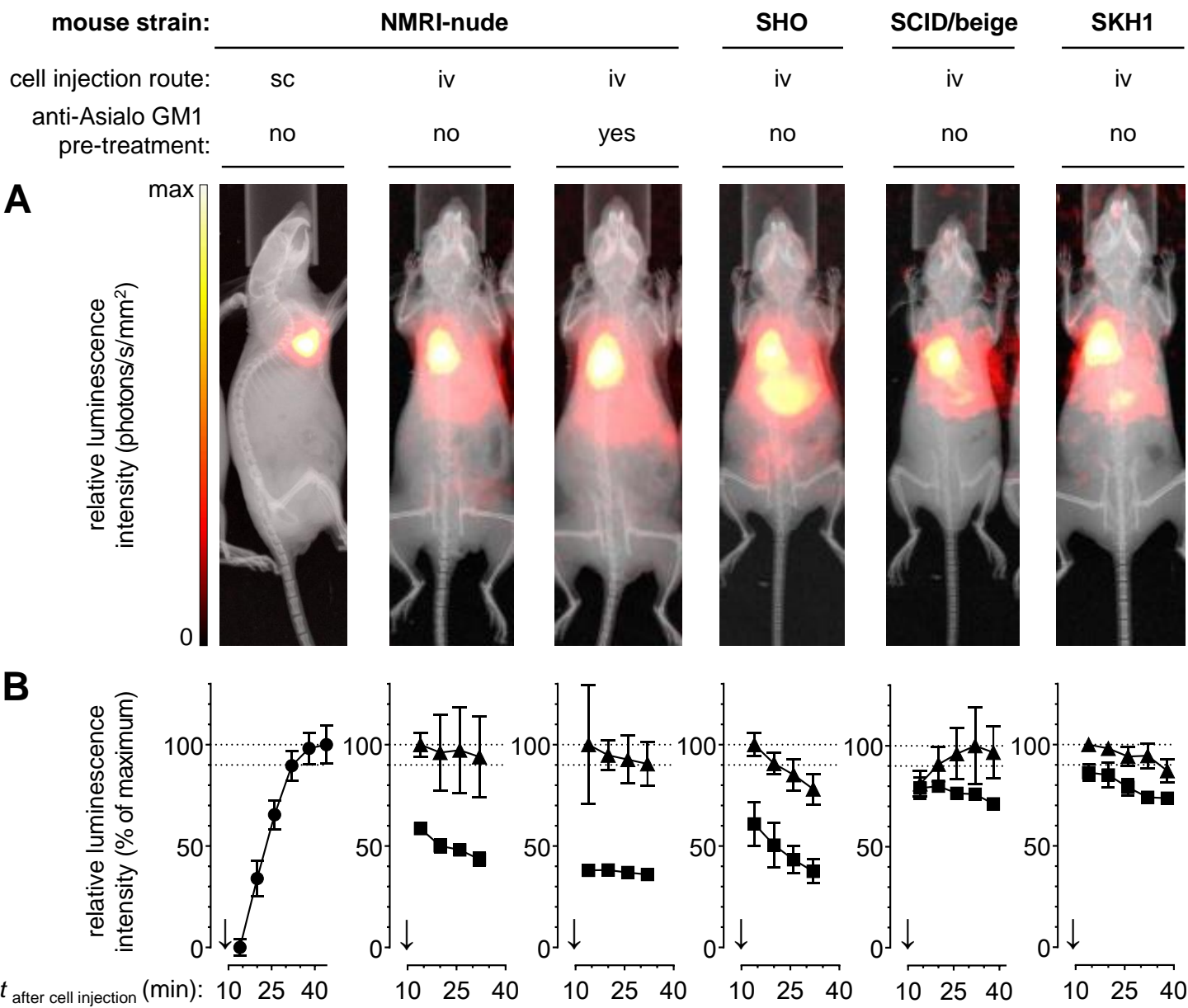

Supplemental Figure 3

Supplement: Supporting Figure 3 [file erc-25-993-s003.pdf]

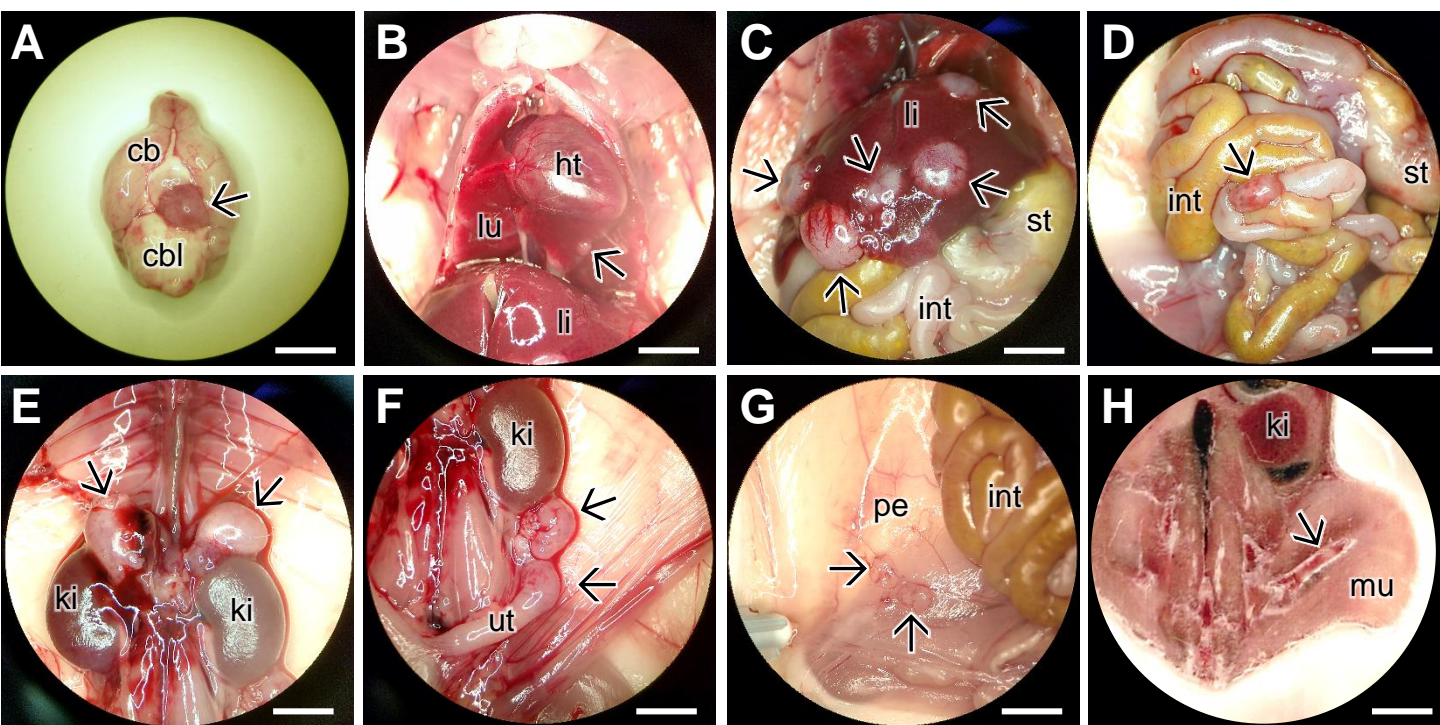

**Supplemental Figure 4**

Supplement: Supporting Figure 4 [file erc-25-993-s004.pdf]

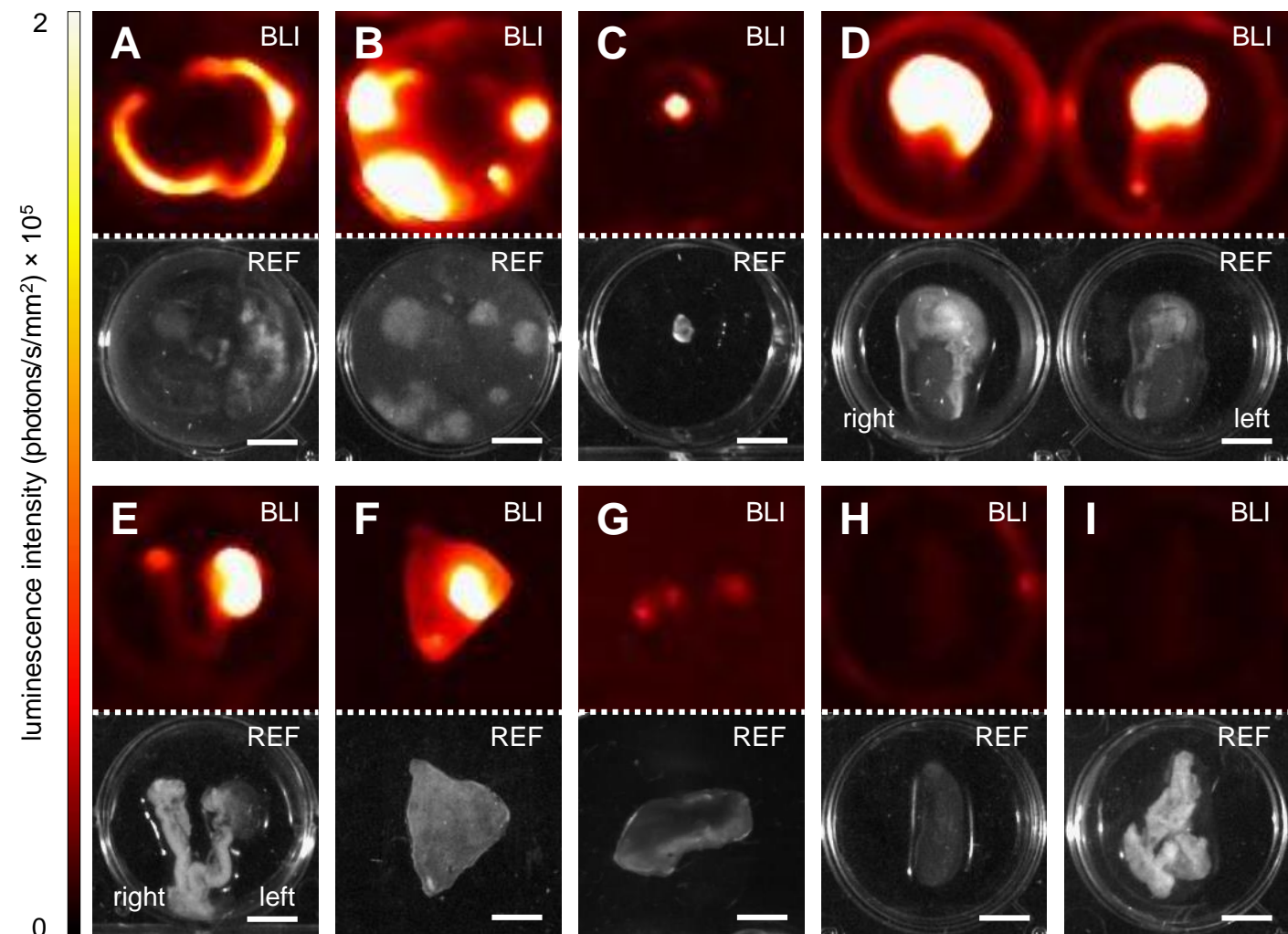

Supplemental Figure 5

Supplement: Supporting Figure 5 [file erc-25-993-s005.pdf]

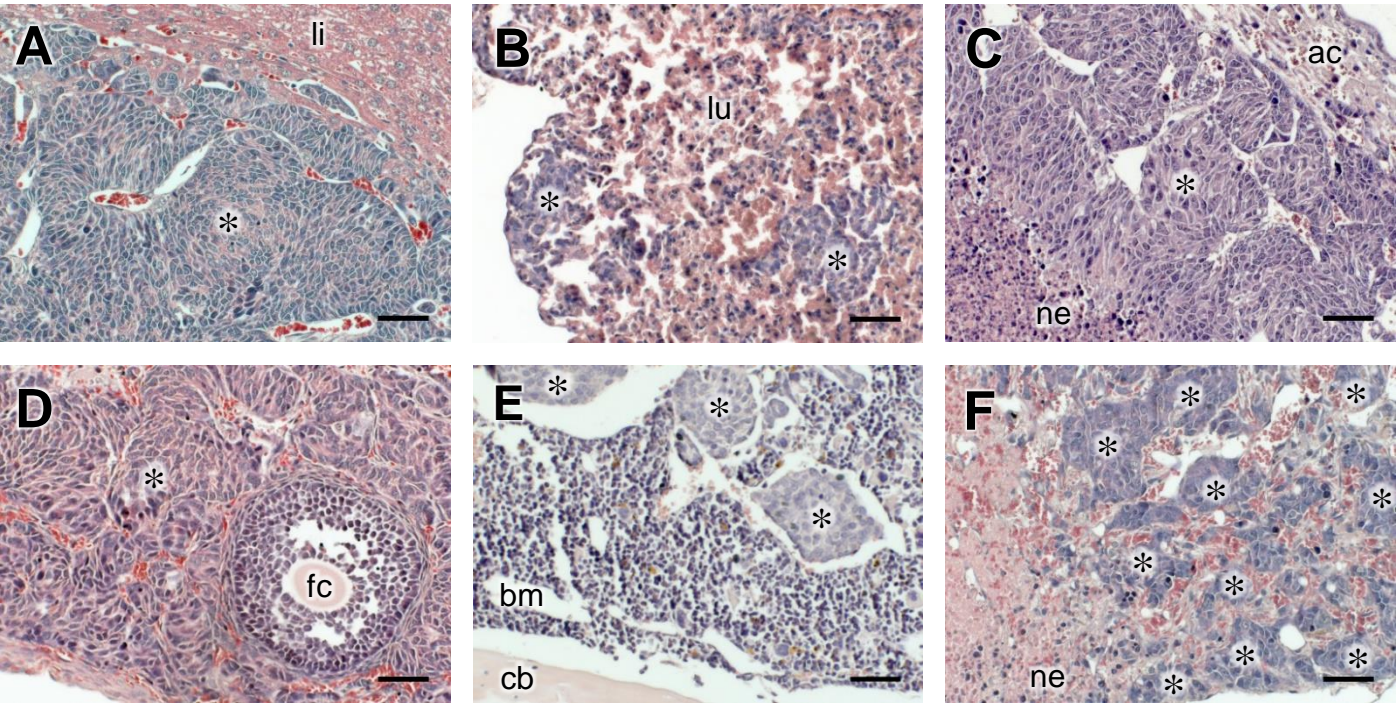

Supplemental Figure 6

Supplement: Supporting Figure 6 [file erc-25-993-s006.pdf]

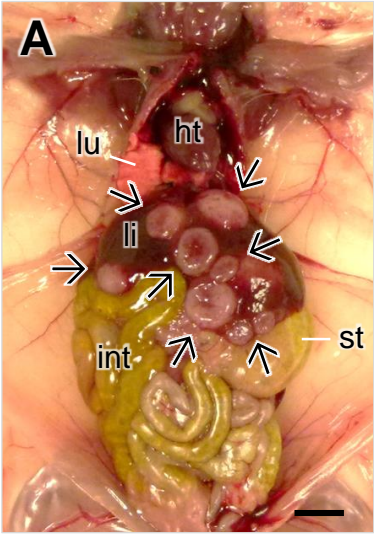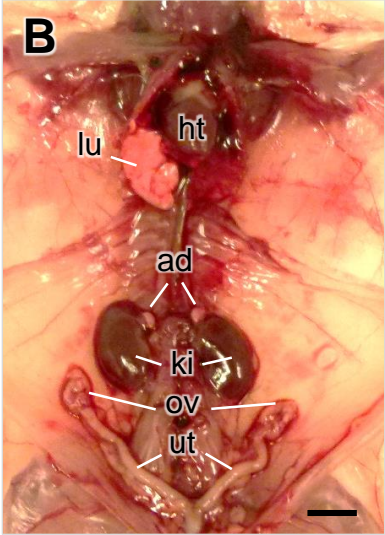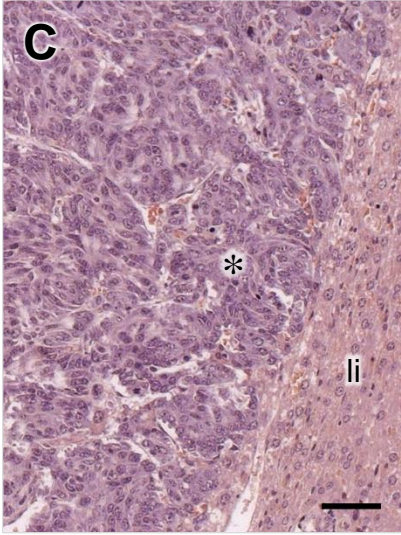

Supplemental Figure 7

Supplement: Supporting Figure 7 [file erc-25-993-s007.pdf]

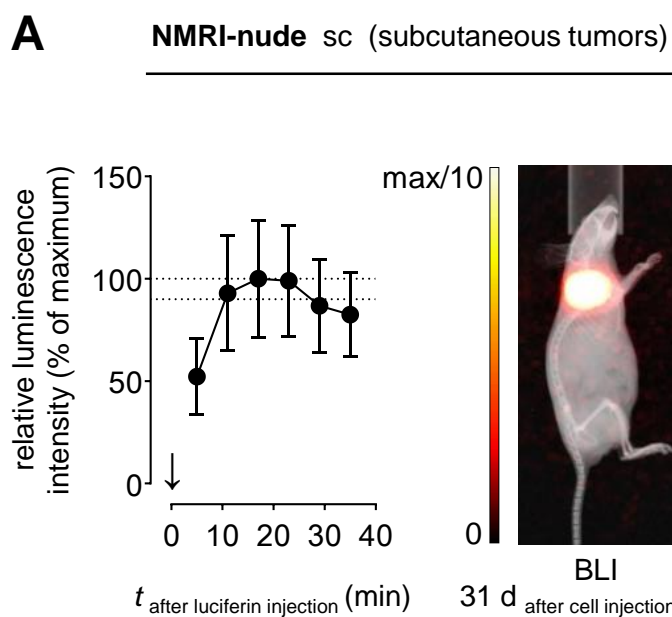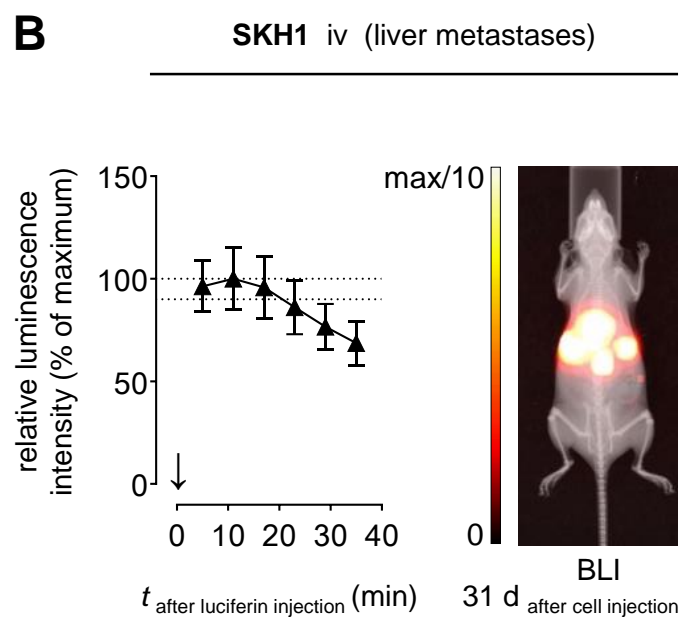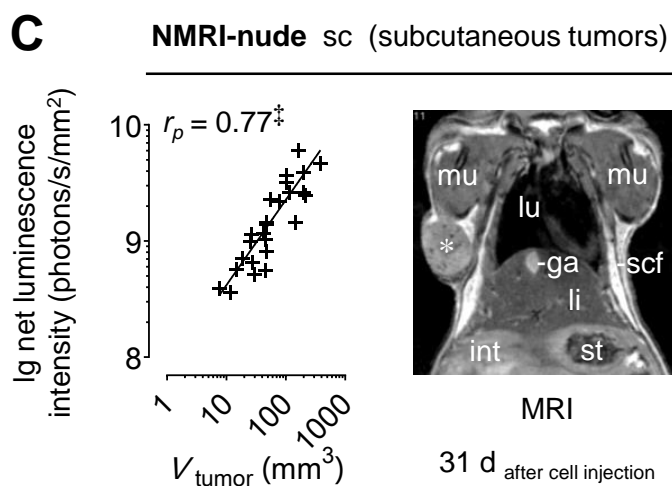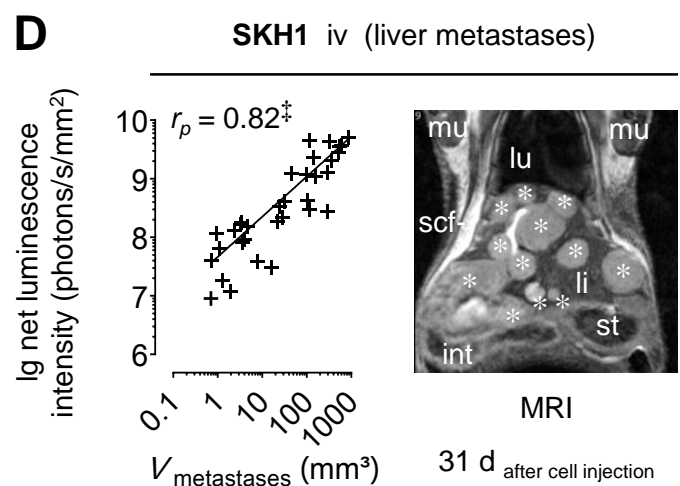

**Supplemental Figure 8**

Supplement: Supporting Figure 8 [file erc-25-993-s008.pdf]

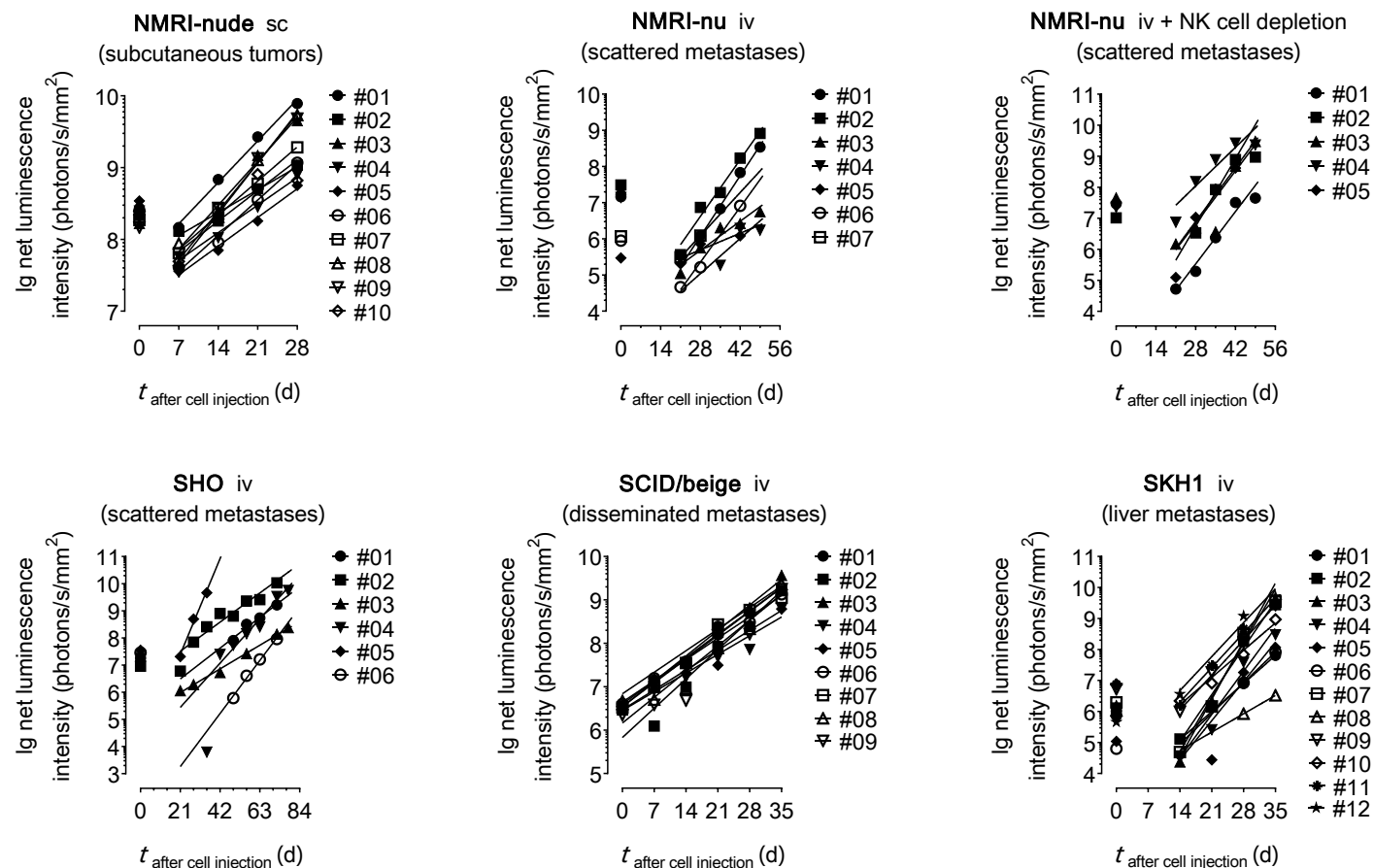

**Supplemental Figure 9**

Supplement: Supporting Figure 9 [file erc-25-993-s009.pdf]
