# Supplementary material for: Strain-specific metastatic phenotypes in pheochromocytoma allograft mice
Source: Endocr Relat Cancer. 2018 Jul 12;25(12):993–1004. doi: 10.1530/ERC-18-0136 (PMC6176113; doi:10.1530/ERC-18-0136)
Supplement: Supplemental material [file erc-25-993-t001.pdf]

Strain-Specific Metastatic Phenotypes in Pheochromocytoma Allograft Mice

Supplemental material

Martin Ullrich[1], Josephine Liers[1,2], Mirko Peitzsch[3], Anja Feldmann[4], Ralf Bergmann[1], Ulrich Sommer[5], Susan Richter[2,3], Stefan R. Bornstein[2,6], Michael Bachmann[2,4,7,8], Graeme Eisenhofer[2,3,6], Christian G. Ziegler[6*], and Jens Pietzsch[1,9*]

[1] Helmholtz-Zentrum Dresden-Rossendorf, Institute of Radiopharmaceutical Cancer Research, Department of Radiopharmaceutical and Chemical Biology, Dresden, Germany
[2] Technische Universität Dresden, Faculty of Medicine Carl Gustav Carus, Dresden, Germany
[3] Technische Universität Dresden, University Hospital Carl Gustav Carus, Institute of Clinical Chemistry and Laboratory Medicine, Dresden, Germany
[4] Helmholtz-Zentrum Dresden-Rossendorf, Institute of Radiopharmaceutical Cancer Research, Department. of Radioimmunology, Dresden, Germany
[5] Technische Universität Dresden, University Hospital Carl Gustav Carus, Institute of Pathology, Dresden, Germany
[6] Technische Universität Dresden, University Hospital Carl Gustav Carus, Department of Internal Medicine III, Dresden, Germany
[7] Technische Universität Dresden, University Hospital Carl Gustav Carus, Universitäts Krebs Centrum (UCC), Tumorimmunology, Dresden, Germany
[8] Technische Universität Dresden, National Center for Tumor Diseases (NCT), Dresden, Germany
[9] Technische Universität Dresden, School of Science, Faculty of Chemistry and Food Chemistry, Dresden, Germany

[*] CGZ and JP shared senior authorship

Corresponding author:

Prof. Jens Pietzsch, PhD, MD
Helmholtz-Zentrum Dresden-Rossendorf
Institute of Radiopharmaceutical Cancer Research
Dept. of Radiopharmaceutical and Chemical Biology
Bautzner Landstraße 400, 01328 Dresden, Germany
Phone: +49-351-2602622, Fax: +49-351-26012622
E-mail: j.pietzsch@hzdr.de

Table of contents

Genetic modification of mouse pheochromocytoma cells 3

Lentiviral vector construction 3

Lentiviral gene transfer 3

In vitro characterization of reporter gene expression in $MPC^{LUC/GZ}$ cells 4

Flow cytometry 4

Confocal laser scanning microscopy 4

Initial $MPC^{LUC/GZ}$ cell distribution in mice 4

Metastasized $MPC^{LUC/GZ}$ allografts in SCID/beige mice 6

Pathology 6

Ex vivo bioluminescence imaging 6

Histopathology 6

Metastasized $MPC^{LUC/GZ}$ allografts in SKH1 mice 7

Pathology and histopathology 7

Monitoring of $MPC^{LUC/GZ}$ allograft progression *in vivo* 7

Multimodal *in vivo* magnetic resonance/bioluminescence imaging 7

Correlation between volume and luminescence intensities of tumors 8

References 9

Genetic modification of mouse pheochromocytoma cells

Lentiviral vector construction

The lentivector p6NST50-luc (Supplemental Figure 1) is a derivative of the previously described p6NST50-MCS, harboring a multiple cloning site (MCS) with unique 5'-*XbaI* and 3'-*HpaI* restriction sites downstream of a spleen focus forming virus (SFFV-U3) promotor (Ho *et al.* 2012). The open reading frame of a firefly luciferase (*luc)* expression cassette (derived from *photinus pyralis*) was ligated into the MCS using 5'-*NheI* and 3'-*PmeI* restriction sites. For selection of successfully gene-modified cells p6NST50-luc harbors a combined enhanced green-fluorescent protein and zeocin resistance (*egfp-zeo)* expression cassette. Bicistronic expression of the selection markers downstream of the transgene stop codon is mediated by an internal ribosomal entry site (IRES) derived from the encephalomyocarditis virus.

Lentiviral gene transfer

Vesicular stomatitis virus G glycoprotein pseudotyped lentiviral particles were generated and viral titers were determined as described elsewhere (Ho *et al.* 2012; Morgenroth *et al.* 2007; Stirnnagel *et al.* 2010). Mouse pheochromocytoma (MPC) cells passage 32 were cultured for 24 h, incubated with the lentiviral particles for 5 h and named $\text{MPC}^{\text{LUC/eGFP-ZEO}}$ cells (abbreviated $\text{MPC}^{\text{LUC/GZ}}$) passage 0.

Although $\text{MPC}^{\text{LUC/GZ}}$ cells and allografts showed efficient and long-lasting luciferase expression in this study, it has been reported that the SFFV-U3 promoter is rapidly silenced by epigenetic remodeling (Warlich *et al.* 2011), an effect that has to be considered during investigations on treatments targeting epigenetics.

In vitro characterization of reporter gene expression in MPC$^{LUC/GZ}$ cells

Flow cytometry

Transgene expression was examined in detached MPC$^{LUC/GZ}$ cell cultures using the flow cytometry system FACSCalibur (BD Biosciences, Franklin Lakes, NJ, USA). Measurement of eGFP reporter protein biosynthesis showed that 91 % of tumor cells were positively luciferase and *egfp* gene-modified after selection with zeocin (Supplemental Figure 2 A).

Confocal laser scanning microscopy

Transgene expression was also examined in adherent MPC$^{LUC/GZ}$ cell cultures using the confocal laser scanning microscope FluoView FW1000 (Olympus, Shinjuku, Tokyo, Japan) confirming eGFP reporter protein biosynthesis in adherent cells exhibiting characteristic MPC cell line-specific growth features such as cluster formation and the outgrowth of neurite-like structures (Supplemental Figure 2 B).

Initial MPC$^{LUC/GZ}$ cell distribution in mice

In vivo bioluminescence imaging (BLI) showed a local accumulation of subcutaneously injected MPC$^{LUC/GZ}$ cells in NMRI-nude mice (reference model). Intravenous tumor cell injection into a tail vein showed a comparable distribution pattern of tumor cells in NMRI-nude, NK cell-depleted NMRI-nude, SHO, SCID/beige, and SKH1 mice, predominantly in lungs, liver, and spleen (Supplemental Figure 3 A).

In order to estimate the duration of MPC$^{LUC/GZ}$ cell accumulation in different models, dynamic BLI was performed in subgroups of three animals starting with injection of luciferin 10 min after tumor cell injection, respectively. In this particular experimental setting, > 90 %

of the maximum luminescence intensity were detected in the subcutaneous reference model (NMRI-nude) later than 32 min after cell injection (Supplemental Figure 3 B). Among intravenously-induced models, > 90 % of the maximum luminescence intensities (strongest signals from lungs) were already detected between 14 and 32 min (NMRI-nude and NK cell-depleted NMRI-nude), between 14 and 20 min (SHO), between 20 and 32 min (SCID/beige), and between 14 and 32 min (SKH1) after cell injection.

Importantly, dynamic *in vivo* BLI data shown here represent a convolution of two parameters: initial distribution of tumor cells and distribution of intraperitoneally injected luciferin. Thus, dynamic BLI only allows for estimating the duration of initial tumor cell accumulation.

In the subcutaneous reference model, slow increase of luminescence intensity at cell injection site is most likely due to slow luciferin distribution from the abdominal cavity to distant subcutaneous tissues. In contrast, intraperitoneally delivered luciferin is much faster available to visceral organs such as lungs and liver. Interestingly, only SCID/beige mice showed increasing luminescence intensities in lungs between 14 and 32 min after cell injection, whereas luminescence intensities decreased immediately in every other metastases model. This observation may be due to prolonged survival of circulating MPC$^{LUC/GZ}$ cells in SCID/beige mice contributing to prolonged organ colonization. Furthermore, luminescence intensities detected in the liver tended to be higher in SCID/beige and SKH1 mice compared to the other metastases models, probably due to a less effective immune response against accumulating MPC$^{LUC/GZ}$ cells particularly in the liver.

Metastasized MPC$^{LUC/GZ}$ allografts in SCID/beige mice

Pathology

For a more detailed description of the term “*disseminated*” MPC$^{LUC/GZ}$ metastases in SCID/beige mice, pathologic observations were photographically documented showing a consistent pattern of multifocal metastases in liver, adrenal glands, bones, lungs, ovaries, and, rarely, also in brain and attached to the peritoneum (Supplemental Figure 4 A-H).

Ex vivo bioluminescence imaging

In order to verify the luciferase-expressing phenotype of MPC$^{LUC/GZ}$ metastases and to validate in particular the methodologic approach for identification of small metastatic lesions, BLI was performed also *ex vivo* in three SCID/beige mice. Dissected organs were incubated in the same D-Luciferin solution used for *in vivo* BLI and imaged immediately (Supplemental Figure 5 A-I). Of note, spleen and pancreas remained free from metastasis.

Histopathology

Microscopic investigations of hematoxylin-eosin-stained target organs of MPC$^{LUC/GZ}$ metastasis performed in three animals showed typical histopathologic features of metastasized PPGL tissue characterized by a trabecular or alveolar pattern with a distinct nest of tumor cells (“zellballen”) often surrounded by structure-supporting sustentacular cells and necrotic regions (Supplemental Figure 6 A-F) as has also been described previously in patient-derived tumor sections (Linnoila *et al.* 1990; Unger *et al.* 1991; van der Harst *et al.* 2000).

Metastasized $MPC^{LUC/GZ}$ allografts in SKH1 mice

Pathology and histopathology

Pathologic observations in SKH1 mice were photographically documented showing that intravenous injection of $MPC^{LUC/GZ}$ cells was predominantly associated with multifocal liver metastases, whereas other digestive organs as well as the entire genito-urinary system remained free from metastases (Supplementary Figure 7 A-B). Microscopy of hematoxylin-eosin-stained liver sections from three animals showed that liver metastases in SKH1 mice exhibited comparable histopathology to liver metastases in SCID/beige mice (Supplemental Figure 7 C).

Monitoring of $MPC^{LUC/GZ}$ allograft progression *in vivo*

In order to validate the performance of BLI for $MPC^{LUC/GZ}$ tumor quantification *in vivo*, correlation between volume (as determined using magnetic resonance imaging, MRI) and luminescence intensities of tumors was analyzed. These particular investigations were selectively performed in sub-cohorts of SKH1 mice bearing liver metastases ($n = 8$) and compared with another sub-cohort of NMRI-nude mice bearing subcutaneous tumors ($n = 6$). Both models were selected with regard to a well-defined localization of lesions allowing for most precise MRI-based measurement of tumor volume.

Multimodal in vivo magnetic resonance/bioluminescence imaging

Optimal time frames for measuring luminescence intensities of tumors were determined from dynamic BLI measurements. Analysis of dynamic image series showed that >90 % of the maximum luminescence intensities were detectable between 11 and 25 min after luciferin

injection in the subcutaneous NMRI-nude reference model and between 5 and 20 min after luciferin injection in the SKH1 liver metastases model (Supplementary Figure 8 A-B).

In order to provide precise tumor volume measurements to be correlated with the luminescence intensities of tumors MRI was performed using the 7 Tesla BioSpin 70/30 scanner (Bruker). At 17, 24, 28, and 31 days after cell injection, T2-weightened images were obtained using a commercial multi-slice multi-echo sequence with an effective echo time of 21.8 ms and a repetition time of 1438 ms at a spatial resolution of 0.2 × 0.2× 0.6 mm and a slice distance of 0.7 mm. Respiratory gating was applied using the control/gating module (SA instruments, Stony Brook, NY, USA). BLI was performed immediately after completion of the MRI scan.

Correlation between volume and luminescence intensities of tumors

The performance of *in vivo* BLI for quantification of $MPC^{LUC/GZ}$ allografts was evaluated in the SKH1 liver metastases model and compared to the subcutaneous NMRI-nude reference model. In both models, correlation analyses between volume and luminescence intensity of tumors showed a significant positive linear relationship, respectively (Supplemental Figure 8 C-D).

These results demonstrate that the *in vivo* BLI approach performed in this study allowed for monitoring the progression of both subcutaneous and metastasized $MPC^{LUC/GZ}$ allografts semi-quantitatively and with comparable accuracy. This approach was based on summating the luminescence intensities from ventral and dorsal images in order to reduce absorption- and attenuation-related inaccuracy resulting from metastases localized at different tissue depths. However, only optical tomography may have the potential to overcome absorption- and attenuation-related limitations of quantitative *in vivo* BLI in the future (Darne *et al.* 2014).

Nevertheless, monitoring the progression of metastasized MPC$^{LUC/GZ}$ allografts in individual animals using *in vivo* BLI (Supplemental Figure 9) provides a fast and precise alternative to other imaging-based metastatic volume measurements such as more time-consuming MRI or radiation-exposing CT scans.

References

Darne C, Lu Y & Sevick-Muraca EM 2014 Small animal fluorescence and bioluminescence tomography: a review of approaches, algorithms and technology update. *Phys Med Biol* **59** R1-64.

Ho YP, Schnabel V, Swiersy A, Stirnnagel K & Lindemann D 2012 A small-molecule-controlled system for efficient pseudotyping of prototype foamy virus vectors. *Mol Ther* **20** 1167-1176.

Linnoila RI, Keiser HR, Steinberg SM & Lack EE 1990 Histopathology of benign versus malignant sympathoadrenal paragangliomas: clinicopathologic study of 120 cases including unusual histologic features. *Hum Pathol* **21** 1168-1180.

Morgenroth A, Cartellieri M, Schmitz M, Gunes S, Weigle B, Bachmann M, Abken H, Rieber EP & Temme A 2007 Targeting of tumor cells expressing the prostate stem cell antigen (PSCA) using genetically engineered T-cells. *Prostate* **67** 1121-1131.

Stirnnagel K, Luftenegger D, Stange A, Swiersy A, Mullers E, Reh J, Stanke N, Grosse A, Chiantia S, Keller H, *et al.* 2010 Analysis of prototype foamy virus particle-host cell interaction with autofluorescent retroviral particles. *Retrovirology* **7** 45.

Unger P, Hoffman K, Pertsemlidis D, Thung S, Wolfe D & Kaneko M 1991 S100 protein-positive sustentacular cells in malignant and locally aggressive adrenal pheochromocytomas. *Arch Pathol Lab Med* **115** 484-487.

van der Harst E, Bruining HA, Jaap Bonjer H, van der Ham F, Dinjens WN, Lamberts SW, de Herder WW, Koper JW, Stijnen T, Proye C, *et al.* 2000 Proliferative index in phaeochromocytomas: does it predict the occurrence of metastases? *J Pathol* **191** 175-180.

Warlich E, Kuehle J, Cantz T, Brugman MH, Maetzig T, Galla M, Filipczyk AA, Halle S, Klump H, Scholer HR, *et al.* 2011 Lentiviral vector design and imaging approaches to visualize the early stages of cellular reprogramming. *Mol Ther* **19** 782-789.
